# Sex Differences in Advanced HIV Disease at Presentation in Care Among People Living With HIV in Latin America and the Caribbean

**DOI:** 10.1002/jia2.70161

**Published:** 2026-08-09

**Authors:** Yanink Caro‐Vega, Jessica Mejía‐Castrejón, Lorena Guerrero‐Torres, Carina Cesar, Claudia P. Cortes, Daisy Maria Machado, Paula M. Luz, Jorge Andrade‐Pinto, Marco Tulio Luque, Eduardo Gotuzzo, Vanessa Rouzier, Jessica L. Castilho, Peter F. Rebeiro, Brenda Crabtree‐Ramírez, for the Caribbean, Central & South American Network for HIV Epidemiology (CCASAnet)

**Affiliations:** ^1^ Departamento De Infectología Instituto Nacional de Ciencias Médicas y Nutrición Salvador Zubirán México City México; ^2^ Fundación Huésped Buenos Aires Argentina; ^3^ Universidad De Chile School of Medicine & Fundación Arriarán Santiago Chile; ^4^ Escola Paulista de Medicina Universidade Federal de São Paulo (UNIFESP) São Paulo Brazil; ^5^ Instituto Nacional de Infectologia Evandro Chagas Fundacao Oswaldo Cruz Rio de Janeiro Brazil; ^6^ Universidade Federal de Minas Gerais Belo Horizonte Brazil; ^7^ Instituto Hondureño de Seguridad Social Tegucigalpa Honduras; ^8^ Instituto De Medicina Tropical Alexander von Humboldt Lima Perú; ^9^ Le Groupe Haïtien d'Etude du Sarcome de Kaposi et des Infections Opportunistes in Port‐au‐Prince (GHESKIO) Port‐au‐Prince Haiti; ^10^ Division of Infectious Diseases Vanderbilt University Medical Center Nashville Tennessee USA

**Keywords:** advanced HIV disease, care continuum, females living with HIV, HIV epidemiology, late presentation, sex disparities

## Abstract

**Introduction:**

Despite an overall recent decline of 28% in the region, AIDS‐related deaths have increased among women in some Latin American countries. Low HIV risk perception among women may contribute to late diagnosis. We compared advanced HIV disease (AHD) at care enrolment between males and females in Latin America and the Caribbean.

**Methods:**

Adults (≥18 years old) from clinical sites in the Central and South America network for HIV epidemiology who enrolled between 2003 and 2022 were stratified by sex‐at‐birth/HIV acquisition group as females, males with male‐to‐male sexual contact and heterosexual males (HMs). We classified location as within a generalized epidemic (GE: Haiti) or a concentrated epidemic (CE: Argentina, Brazil, Chile, Honduras and Mexico). We estimated the probability of AHD (AIDS diagnosis or CD4<200 cells/µL) at enrolment in care using logistic regression models, stratifying by sex‐at‐birth/acquisition group and CE/GE setting, adjusting for age, education level and calendar year of enrolment.

**Results:**

Of 47,548 people living with HIV, 51% were from GE sites (58% females), and 49% were from CE sites (20% females, 22% HMs and 58% males with male‐to‐male sexual contact). In GE, 27.7% of persons presented with AHD (23.5% of females; 33.3% of males), while in CE, 44.9% of persons presented with AHD (37.4% of males with male‐to‐male sexual contact, 47.8% of females and 61.8% of HMs). Males in GE had higher odds of AHD compared with females (aOR: 1.48 [95% CI: 1.40–1.57]). The odds of AHD decreased from 2003 to 2022 (aOR: 0.29 [95% CI: 0.26–0.33] in GE and aOR: 0.32 [95% CI: 0.29–0.36] in CE). In sex‐at‐birth/acquisition stratified models, in CE sites, odds decreased more in males with male‐to‐male sexual contact (aOR: 0.22 [95% CI: 0.19–0.25]) than in females (aOR: 0.57 [95% CI: 0.46–0.79]) and HMs (aOR: 0.47 [95% CI: 0.38–0.58]) in 2022 versus 2003.

**Conclusions:**

In CE sites, the slight decreasing trend of AHD at enrolment among females and HMs contrasts with the steeper decline among males with male‐to‐male sexual contact. In the GE site, risk of AHD at enrolment decreased in both males and females over time. Interventions to increase awareness, improve HIV testing, and hasten linkage to care among females and HMs are needed in CE settings in addition to addressing structural and social barriers.

## 1 Introduction

According to the Joint United Nations Programme on HIV/AIDS (UNAIDS), 53% of all people living with HIV (PWH) globally were women and girls, and 45% of all new HIV acquisitions are among women and girls [1]. While the number of new acquisitions and AIDS‐related deaths has decreased more rapidly in women compared to men across all age groups, and women generally experience better HIV outcomes, with 91‐83%–78% meeting the 95‐95‐95 targets versus 83‐72%–67% in men, significant regional differences persist [2]. In Latin America and the Caribbean, despite a 28% decline overall in deaths from 2010 to 2023, the number of AIDS‐related deaths among women has increased in Costa Rica, El Salvador, Mexico, Panama, Paraguay and Peru [3]. Up to 2024, the decline in overall deaths from 2010 was reported at 31%, but the figure among women was not updated [4].

Around the world, policies for monitoring and controlling HIV have been tailored to the specific needs of each country based on HIV prevalence patterns and, in some settings, funding structures such as donor‐supported programmes [5]. Some countries are considered to have a generalized epidemic (>1% HIV prevalence in the total population), and others have a concentrated epidemic (total prevalence <1% but significantly higher in key populations) [6].

Most of the countries in Latin America and the Caribbean have concentrated epidemics; consequently, policies have largely focused on key populations (including men who have sex with men, sexual workers, transgender people), while females and girls are perceived as being at lower risk of HIV acquisition and are offered fewer screening and prevention services [7]. In addition, gender inequalities persist in Latin America, including gender‐based violence, discrimination, marginalization, poverty and denial of rights, as well as lack of sexual and reproductive health services [8]. Domestic or sexual violence, having a stable partner [9], older age, lower educational attainment and unemployment [10, 11] are also associated with a higher risk of late HIV diagnosis in females.

Late diagnosis of HIV and advanced HIV disease (AHD) are public health challenges associated with increased risk of death [12], interruptions in care, higher healthcare costs [13], increased HIV transmission [14], disability [15], and eventual non‐infectious comorbidities and multimorbidity [16]. However, in‐depth analyses of females with HIV in Latin America and the Caribbean to understand sex at birth differences in AHD risk and outcomes remain limited to date, limiting the development of evidence‐based, person‐centred approaches. To address this gap, our study compared trends in the proportion of males and females living with HIV with AHD at enrolment in care across Latin America and the Caribbean, in the context of concentrated and generalized epidemics.

## 2 Methods

### 2.1 Study Population

We included PWH ≥18 years old who enrolled in care from 1 January 2003, until 31 December 2022, at clinical sites of the Caribbean, Central and South America network for HIV epidemiology (CCASAnet). CCASAnet is a multinational observational cohort collaboration of HIV clinical sites in seven Latin American and Caribbean countries: Centro Médico Huésped, Buenos Aires, Argentina (CMH‐Argentina); Instituto Nacional de Infectologia Evandro Chagas, Fundação Oswaldo Cruz, Rio de Janeiro, Brazil (FC‐Brazil); Fundación Arriarán, Santiago, Chile (FA‐Chile); Le Groupe Haïtien d'Etude du Sarcome de Kaposi et des Infections Opportunistes, Port‐au‐Prince, Haiti (GHESKIO‐Haiti); Instituto Hondureño de Seguridad Social and Hospital Escuela, Tegucigalpa, Honduras (IHSS/HE‐Honduras); Instituto Nacional de Ciencias Médicas y Nutrición Salvador Zubirán, Mexico City, México (INCMNSZ‐Mexico); and Instituto de Medicina Tropical Alexander von Humboldt, Lima, Perú (IMTAvH‐Peru) [17]. The data used in this study were collected between 2003 and 2022. Data from Peru were available until December 2019.

We included data from males and females in all sites, excluding those self‐reported as transgender because the information has only recently been consistently collected and the sample size is still small. We also excluded persons from concentrated epidemics with a “non‐sexual acquisition,” such as perinatal, injection drug use, haemophilia related and those reported as “other mode” or “unknown mode” from the main analysis. This group represents only 5% of the total population in the CCASAnet cohort, with more than 80% classified as perinatal HIV acquisition. We excluded this subgroup because their determinants of entry into care are likely different from those shaping enrolment among individuals with sexually acquired HIV, which is the focus of this analysis. Our disaggregation by sex does not imply any assumption regarding the directionality of HIV transmission between sexual partners.

### 2.2 Study Population, Outcomes and Definitions

AHD was defined as the closest CD4 cell count <200 cells/µL or any AIDS‐defining event before or at enrolment (180 days before until 90 days after enrolment date) [18]. This time window was agreed between clinicians and statisticians to keep the maximum amount of data without affecting the CD4 count and AHD association, consistent with our previous approaches [19, 20, 21].

We stratified sites by epidemic type according to the World Health Organization's definition [22] of generalized and concentrated epidemics. Haiti was classified as generalized, while Argentina, Brazil, Chile, Honduras, Mexico and Peru were classified as concentrated. Our previous studies highlight several differences in demographics and care outcomes by HIV acquisition [10, 11, 23]. We categorized individuals by sex at birth, as females and males in the generalized epidemic site and three groups in the concentrated epidemic sites combining sex at birth and HIV acquisition variables: females, heterosexual males (HMs) and males with male‐to‐male sexual contact. Females only reported sexual acquisition without any additional specification regarding the sex of their sexual partners.

### 2.3 Statistical Analyses

We described and compared general sociodemographic and clinical characteristics at enrolment by sex‐at‐birth/HIV acquisition and epidemic type groups. We used medians and interquartile ranges for continuous variables as CD4 count, age and time since HIV diagnosis. We estimated frequencies and proportions by category for educational level (categorized as less than primary, secondary, university and unknown). We used the Kruskal−Wallis test and chi‐square test, respectively, to compare characteristics by sex‐at‐birth/HIV acquisition group in each epidemic type.

We estimated the proportion of AHD by group of sex‐at‐birth/HIV acquisition and epidemic type in the study period. We employed several logistic models to examine the trend of the probability of AHD at enrolment by sex‐at‐birth/HIV acquisition in each epidemic type, fitting by age, educational level and calendar year. Age and year of enrolment were modelled using splines with three nodes. This methodology allowed us to avoid linear assumptions between the outcome (AHD probability) and continuous variables, and to estimate the risks for specific values of the variables [19]. Results are shown for ages 30, 40 and 50 years old; and for the calendar years 2003, 2010 and 2022; using the first value of each variable as the reference category to make the comparisons between values easier.

First, to compare the odds of AHD between sex‐at‐birth/HIV acquisition groups, we conducted two logistic models for each epidemic setting using sex‐at‐birth/HIV acquisition group as the main exposure. Then, we fit five logistic models for group of sex‐at‐birth/HIV acquisition in each epidemic type, to identify potential differences in the association of other factors with the odds of AHD by group and to visualize the trend over time. We described the characteristics of the models we fitted in Table S4.

In a sensitivity analysis, we included participants with an unknown mode of HIV acquisition in the concentrated epidemic models. First, we fit a logistic model to compare the odds of AHD between groups, including “unknown” as a category of HIV acquisition. Then, we kept females with this unknown data in the female group, and we imputed the mode of HIV acquisition in males with unknown mode of acquisition, using chained equations with 10 replications. We pooled the results using Rubin's rules [19]. We used a logistic model to analyse the trend of AHD over time and to compare the risk of AHD in females and HMs with males with male‐to‐male sexual contact group in the imputed dataset. The analyses were performed in Mexico using R, version 4.3.1.

### 2.4 Ethical Approval

Institutional review board (IRB) approval was obtained locally for each participating site and the CCASAnet data coordinating centre at Vanderbilt University Medical Center, Nashville, TN, USA. At all participating sites, except IMTAvH‐Peru, ethical regulations and policies permit retrospective analysis of de‐identified clinical data without informed consent, provided that the research is approved by an IRB or appropriately constituted ethics committee. At IMTAvH‐Peru, patients provide informed consent at the time of enrolment to allow the use of de‐identified clinical data for research studies.

## 3 Results

### 3.1 General Characteristics

**FIGURE 1:**
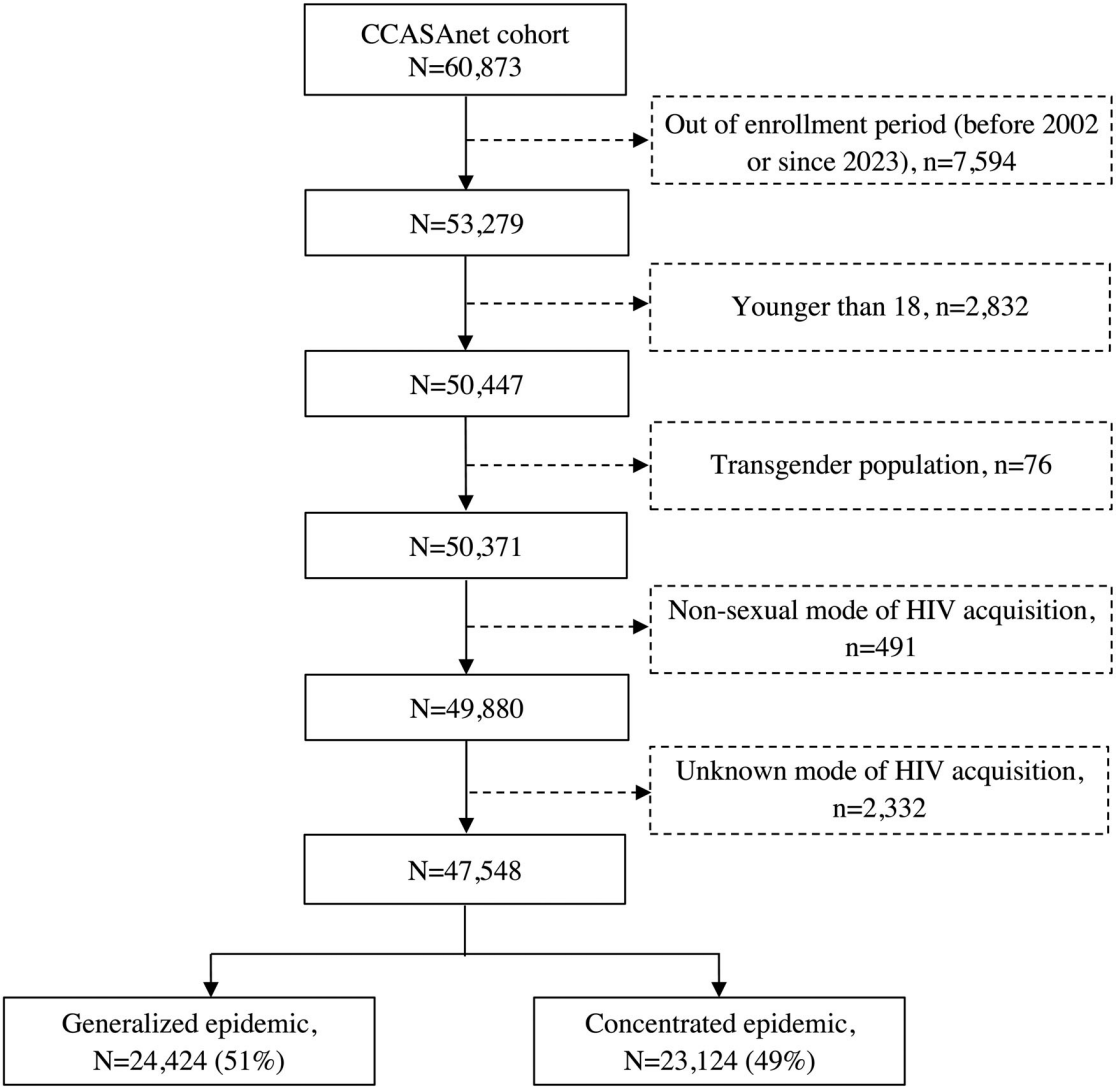
Flowchart of cohort creation. Solid lines represent included and dotted lines represent the excluded individuals.

We included 47,548 participants, 24,424 (51%) in the generalized epidemic and 23,124 (49%) in the concentrated epidemic settings (Figure 1). Overall, 18,788 (39%) were females, 15,338 (32%) HMs and 13,432 (28%) males with male‐to‐male contact. Of all, 17,136 (36%) individuals had AHD at enrolment: 6762 (39.5%) in generalized epidemic and 10,374 (60.5%) in the concentrated epidemic sites. Of all individuals with AHD, 5553 (32%) were females.

### 3.2 Generalized Epidemic

**FIGURE 2:**
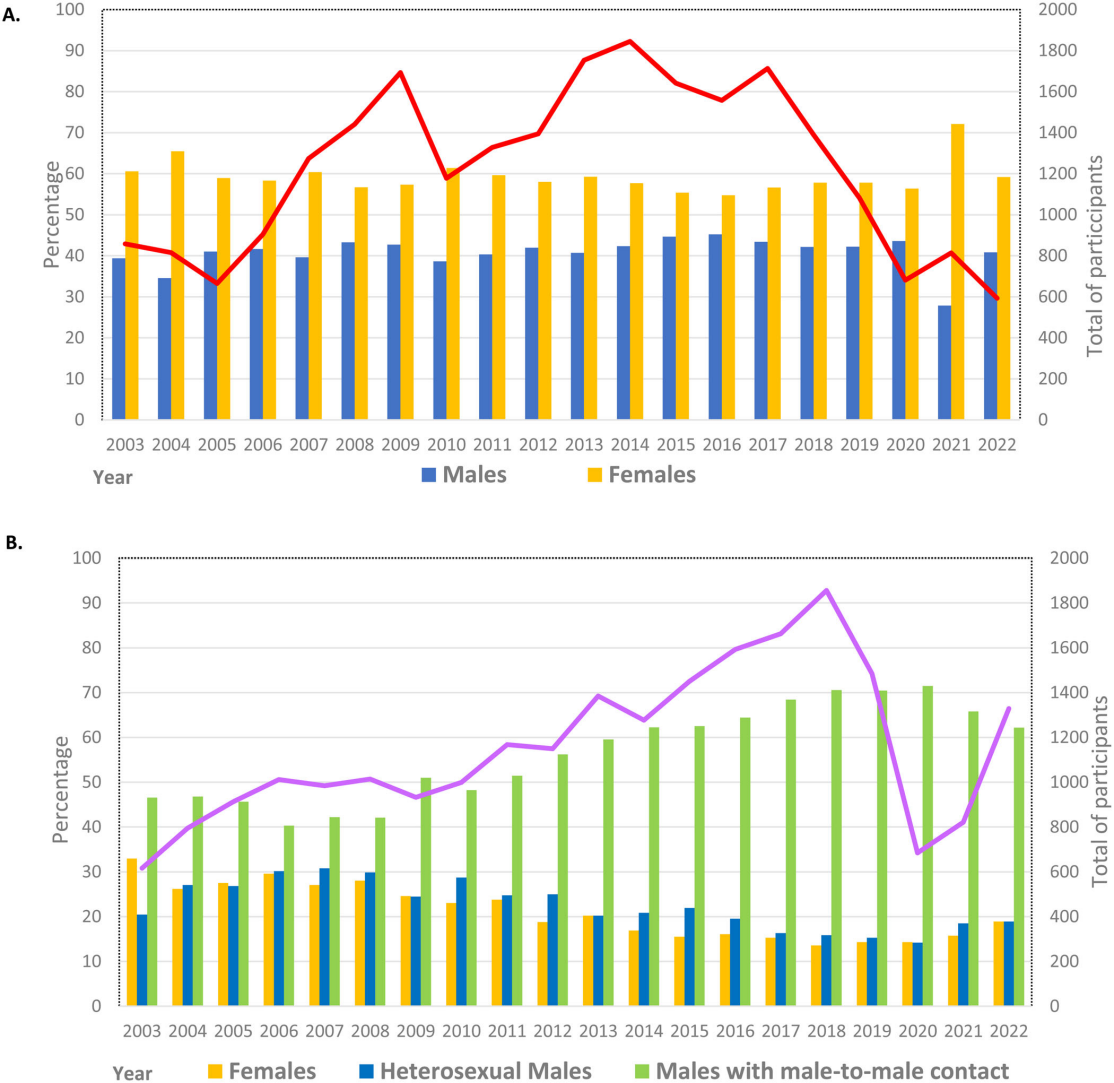
Distribution by sex of participants in the cohort by epidemic type and calendar year. Continuous purple lines correspond to the total number of participants enrolled in care by calendar year. (A) Generalized epidemic. (B) Concentrated epidemics. *Note*: Solid purple lines represent the total number of participants enrolled in care by calendar year and are shown on the right‐hand axis in each figure.

In Haiti, 58% of PWH included were females, with a relatively stable proportion of females over time (61% of all enrolees in 2003 and 60% in 2022) (Figure 2, Panel A). Compared to males, females were younger (33.8 vs. 38.5 years of age); had a higher median of CD4 count at enrolment (266 vs. 203 cells/µL) and were less likely to have a high education level (1.8% of females vs. 5.2% of males with university level). Overall, AHD prevalence at enrolment was 23.6% in females and 33.3% in males (Table 1).

**TABLE 1:** Characteristics at enrolment in care by type of HIV epidemic and group of sex‐at‐birth and sex‐at‐birth/HIV acquisition.

HIV epidemic		Generalized epidemic 24,424 (51%)		Concentrated epidemic 23,124 (49%)		
Variable	Group	Females	Males	Females	Heterosexual males	Males with male‐to‐male sexual contact
***N* (%)**		14,141 (58)	10,283 (42)	4637 (20)	5055 (22)	13,423 (58)
**Days since HIV diagnoses to enrolment,** median (IQR)^a^		0 (0–20)	0 (0–22)	60 (10–643)	43 (7–320)	84 (21–640)
**Age in years**, median (IQR) ^a^		33.82 (27.2–42.4)	38.51 (31.4–46.6)	34.58 (27.9–43.1)	36.9 (29.7–45.9)	31.04 (25.8–38.3)
**CD4 count, cells/mL**, median (IQR) ^a^		266 (128–443)	203 (74–342)	245 (96–458)	164 (54–343)	315 (140–501)
Missing CD4 count, *n* (%)		7460 (52.7)	5103 (49.6)	356 (7.7)	429 (8.5)	830 (6.2)
**Education level,** *n* (%)^a^						
Less than primary		3242 (23.1)	1641 (16.1)	1087 (23.4)	928 (18.3)	746 (5.5)
Secondary		10,002 (70.7)	7523 (73.1)	2334 (50.3)	2533 (50.1)	5495 (40.9)
University		259 (1.8)	540 (5.2)	718 (15.5)	1179 (23.3)	6057 (45.1)
Unknown		628 (4.4)	579 (5.6)	498 (10.7)	415 (8.2)	1134 (8.4)
**Advanced HIV disease**, *n* (%)^a^		3333 (23.5)	3429 (33.3)	2220 (47.8)	3126 (61.8)	5028 (37.4)

^a^ *p*‐value<0.01 in the comparison between groups in each epidemic type.

In the multivariable analyses, we found that males had higher odds of AHD than females in the general epidemic site: aOR: 1.48 (95% CI: 1.40–1.57). Odds of AHD at enrolment was also associated with older ages (aOR: 1.44 [95% CI: 1.37–1.53] for people at 50 years old vs. people at 30 years old), education level (though not statistically significant) and earlier calendar year (2022 compared with 2003 aOR: 0.29 [95% CI: 0.26–0.33]) (Table 2).

**TABLE 2:** Associated characteristics with advanced HIV disease at enrolment in care in generalized epidemic overall and by sex at birth.

		Model without stratification by sex at birth aOR (95% CI)	Stratified by sex at birth models^a^	
Variable			Females aOR (95% CI)	Males aOR (95% CI)
**Sex at birth**	Males	1	—	—
	Females	0.67 (0.64–0.72)	—	—
**Age at enrolment**	30	1	1	1
	40	1.39 (1.36–1.44)	1.43 (1.37–1.50)	1.31 (1.23–1.40)
	50	1.44 (1.37–1.53)	1.53 (1.41–1.65)	1.34 (1.23–1.45)
**Education level**	Less than primary	0.93 (0.87–1.01)	0.91 (0.83–1.00)	0.95 (0.84–1.07)
	Secondary	1	1	1
	University	1.09 (0.94–1.28)	1.33 (1.00–1.75)	1.01 (0.84–1.22)
	Unknown	1.13 (0.98–1.30)	1.17 (0.96–1.37)	1.09 (0.90–1.32)
**Calendar year of enrolment**	2003	1	1	1
	2010	0.90 (0.83–0.99)	0.89 (0.78–1.00)	0.93 (0.82–1.06)
	2022	0.29 (0.26–0.33)	0.28 (0.24–0.33)	0.30 (0.26–0.36)

^a^ Overall association results from a logistic model without sex at birth stratification. Results of multivariable logistic models for AHD in each group of sex at birth. Age and calendar year were modelled as continuous variables using splines. Models 1−3 in Table S4.

In the multivariable models stratified by sex at birth, age was similarly associated with AHD risk in females (aOR: 1.43 [95% CI: 1.37–1.50] for 40 vs. 30 years old) and in males (aOR: 1.31 [95% CI: 1.23–1.40]). The AHD risk trend did not significantly decrease during the study period for either females or males; aOR: 0.89 [95% CI: 0.78–1.00] in 2010 versus 2003 among females and aOR: 0.93 [95% CI: 0.82–1.06] in 2010 versus 2003 in males. Similarly, education level was not significantly associated with risk of AHD in females (aOR: 0.91 [95% CI: 0.83–1.00] comparing less‐than‐primary vs. secondary levels) nor males (aOR: 0.95 [95% CI: 0.84–1.07]) (Table 2).

### 3.3 Concentrated Epidemics

In concentrated epidemic settings, 20% of all PWH were females, 22% were HMs and 58% were males with male‐to‐male sexual contact (Table 1). The percentage of females in concentrated epidemic sites over time ranged from 33% in 2003 to 19% in 2022, compared with 20% to 19% HMs in the same period (Figure 2, Panel B). Overall, females were older than males with male‐to‐male sexual contact at enrolment (median age of 34.6 vs. 31.0 years) but younger than HMs (36.9 years) (*p*<0.01). Compared with males with male‐to‐male sexual contact, females and HMs had lower CD4 counts at enrolment: median of 245 cells/µL among females, 164 cells/µL among HMs and 315 cells/µL among males with male‐to‐male sexual contact. AHD at enrolment was prevalent in 47.8% of females, 61.8% of HMs and 37.4% of males with male‐to‐male sexual contact. Time since HIV diagnosis to enrolment was lowest in HMs: 43 days compared to 60 days among females and 84 days among males with male‐to‐male sexual contact.

In the multivariable model, compared with males with male‐to‐male sexual contact, females and HMs were at increased odds of AHD at enrolment: aOR: 1.12 (95% CI: 1.04–1.20) and aOR: 2.00 (95% CI: 1.86–2.14), respectively. Higher odds of AHD was also associated with older age (aOR: 1.47 [95% CI: 1.39–1.56] for 50 vs. 30 years old), lower education (aOR: 1.31 [95% CI: 1.19–1.43] for less‐than‐primary vs. secondary level), earlier calendar year of enrolment (aOR: 0.76 [95% CI: 0.70–0.83] for 2010 vs. 2003 and aOR: 0.32 [95% CI: 0.29–0.36] for 2022 vs. 2003) (Table 3).

**TABLE 3:** Associated characteristics with advanced HIV disease at enrolment in care in the study period stratified by group of sex‐at‐birth/HIV acquisition in concentrated epidemics.

Variable	Group	Model without stratification by sex‐at‐birth/HIV acquisition group	Stratified by sex‐at‐birth/HIV acquisition group models^a^		
			Females	Heterosexual males	Males with male‐to‐male sexual contact
**Sex at birth**	Males with male‐to‐male sexual contact	1	—	—	—
	Females	1.12 (1.04–1.20)	—	—	—
	Heterosexual males	2.00 (1.8–2.15)	—	—	—
**Age at enrolment**	30	1	1	1	1
	40	1.44 (1.42–1.49)	1.47 (1.37–1.57)	1.29 (1.19–1.45)	1.44 (1.38–1.49)
	50	1.47 (1.39–1.56)	1.56 (1.39–1.75)	1.31 (1.19–1.39)	1.45 (1.31–1.59)
**Education level**	Less than primary	1.31 (1.19–1.43)	1.37 (1.18–1.59)	1.28 (1.09–1.50)	1.71 (1.46–2.01)
	Secondary	1	1	1	1.33 (1.23–1.44)
	University	0.76 (0.72–0.81)	0.81 (0.68–0.96)	0.71 (0.62–0.82)	1
	Unknown	0.97 (0.81–1.07)	1.22(1.00–1.49)	0.94 (0.76–1.17)	1.13 (0.99–1.29)
**Calendar year of enrolment**	2003	1	1	1	1
	2010	0.76 (0.70–0.83)	0.66 (0.55–0.79)	0.99 (0.82–1.20)	0.70 (0.63–0.78)
	2022	0.32 (0.29–0.36)	0.57 (0.46–0.79)	0.47 (0.38–0.58)	0.22 (0.19–0.25)

*Note*: Overall association results from a logistic model without sex‐at‐birth/HIV acquisition stratification.

^a^ Results of multivariable logistic models for AHD in each group of sex‐at‐birth/HIV acquisition. Age and calendar year were modelled as continuous variables using splines. Models 4–7 in Table S4.

In adjusted models stratified by sex/HIV acquisition, older age at enrolment was similarly associated with higher odds of AHD in all groups (40 vs. 30 years old aOR: 1.47 [95% CI: 1.37–1.57] in females, aOR: 1.29 [95% CI: 1.19–1.45] in HMs; and aOR: 1.44 [95% CI: 1.38–1.49] in males with male‐to‐male sexual contact). Less education was associated with higher odds of AHD for all sex‐at‐birth/HIV acquisition groups: less than primary compared to secondary level in females: aOR: 1.37 (95% CI: 1.18–1.59) and HMs: aOR: 1.28 (95% CI: 1.09–1.50); and less than primary compared with university level in males with male‐to‐male sexual contact: aOR: 1.71 (95% CI: 1.46–2.01) (Table 3).

The calendar year had a heterogeneous association with AHD. Among males with male‐to‐male sexual contact, there was a significant decrease in 2010 compared to 2003: aOR: 0.70 [95% CI: 0.63–0.78]. Among females, a lower odds of AHD was observed in 2010 compared with 2003 (aOR: 0.66 [95% CI: 0.55–0.79]), whereas no change was observed for the same period in HMs (aOR: 0.99 [95% CI: 0.82–1.20]). The odds of AHD significantly decreased for all three groups in 2022 compared with 2003: aOR: 0.57 (95% CI: 0.46–0.79) in females; aOR: 0.47 (95% CI: 0.38–0.58) in HMs and aOR: 0.22 (91% CI: 0.19–0.25) in males with male‐to‐male sexual contact (Table 3).

### 3.4 Trend of AHD by Sex‐at‐Birth/HIV Acquisition Risk Group and Epidemic

**FIGURE 3:**
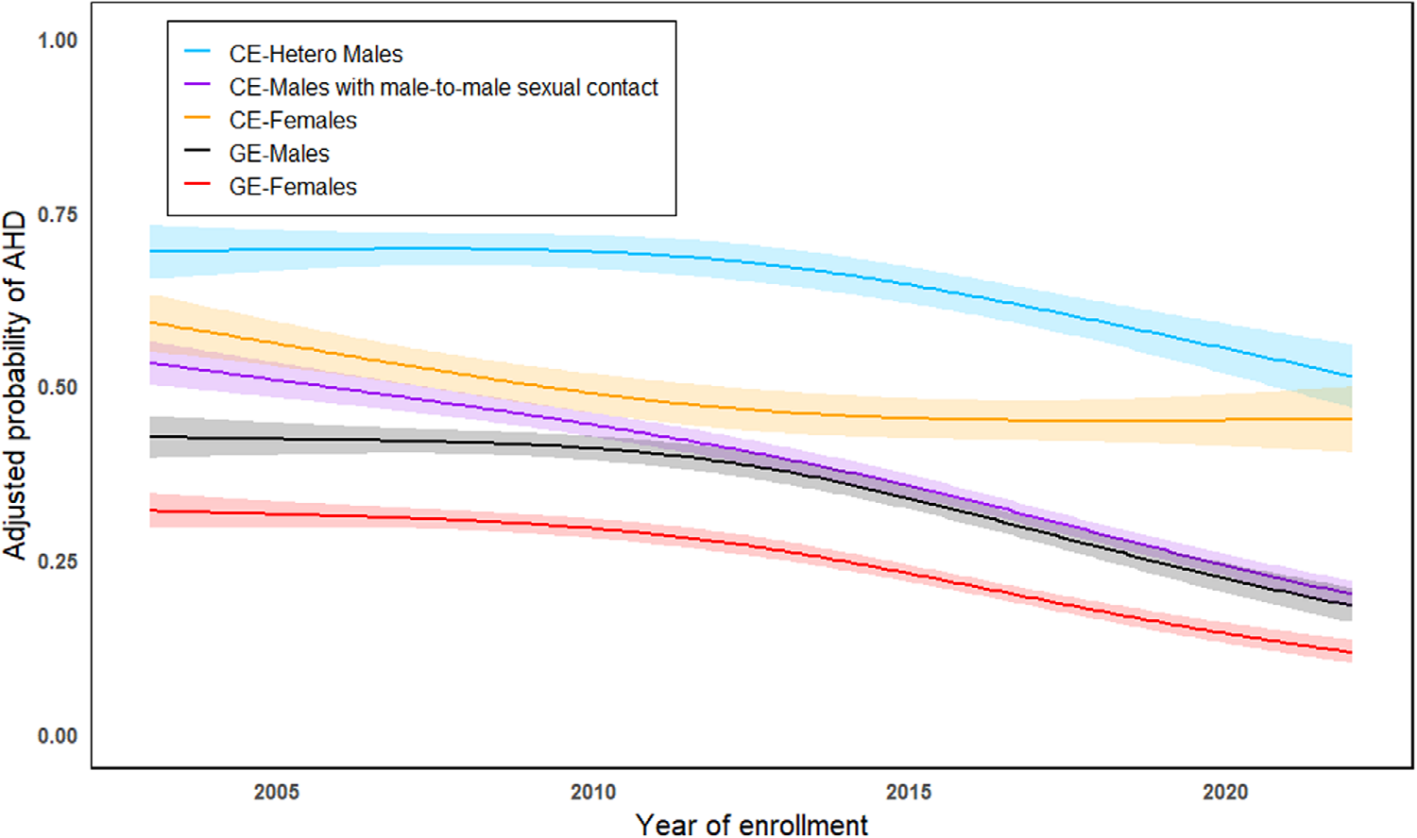
Adjusted probability of advanced HIV disease (AHD) at enrolment over time by sex at birth and HIV acquisition groups and epidemic setting. *Note*: Curves are predictions from five multivariable logistic models of advanced HIV disease (AHD, AIDS diagnosis or CD4<200 cells/µL) at enrolment, stratified by sex at birth (females and males) and HIV acquisition group (females, males with male‐to‐male sexual contact; and HMs: heterosexual males) and epidemic type (CE, concentrated epidemic, GE, generalized epidemic), including year of enrolment, education level and age at enrolment. Models 2, 3, 5–7 in Table S4.

The adjusted probability of AHD across time by sex‐at‐birth/HIV acquisition groups from stratified models is shown in Figure 3. In generalized epidemic, the estimated probability of AHD decreased over time in both males and females. In concentrated epidemics, the estimated probability was stable and slightly increasing in the last years for females but decreasing for both HMs and males with male‐to‐male sexual contact, particularly in males with male‐to‐male sexual contact.

### 3.5 Concentrated Epidemics: Sensitivity Analyses

We included 541 females and 1791 males with unknown HIV acquisition group as an additional category, and estimated the trend of AHD for them, compared to original groups (Figure S1). The trend of AHD for the unknown HIV acquisition group showed a slight decrease in the probability around 2010 with a more recent increase, similar to the trend among females (Figure S1). After the imputation process, we found that females and HMs, compared to males with male‐to‐male sexual contact, had higher odds of AHD, aOR: 1.15 (95% CI: 1.07–1.25) and aOR: 1.96 (95% CI: 1.81–2.11), respectively (Table S1). We also observed a trend of lower odds of AHD over time: aOR: 0.73 (95% CI: 0.68−0.79) for 2010 compared with 2003 and aOR: 0.36 (95% CI: 0.32−0.39) for 2022 compared with 2003 (Table S1 and Figure S1). The results of the stratified models before and after the imputation process are shown in Tables S2 and S3.

## 4 Discussion

Our study revealed that more than a third of PWH from the CCASAnet sites are enrolled into care with AHD, and that 32% of those with AHD were females. The prevalence of AHD at enrolment in care over time was higher in sites in concentrated epidemics than in sites with generalized epidemic. While the adjusted odds of AHD decreased from 2003 to 2022 in the region, in both epidemic settings, but particularly in males with male‐to‐male sexual contact at sites within concentrated epidemic settings, smaller declines were observed in HMs and females.

Our study is consistent with others from Latin America that have shown persistently high rates of late HIV diagnosis, particularly in concentrated epidemic settings. Other studies have reported more than 70% of PWH with late presentation at HIV diagnosis [24, 25]. On the other hand, the prevalence of AHD at Antiretroviral Therapy initiation reported in Caribbean countries with generalized epidemics is similar to our results and lower compared with estimations in concentrated epidemics [11]. Although the rates of AHD at Antiretroviral Therapy initiation have declined [26], AHD at HIV diagnosis continues to be a persistent challenge in the Caribbean region [27]. We are not able to directly compare our findings with those studies because none disaggregated their results by sex at birth, and some analysed AHD at different times of HIV care.

Though males in Haiti (generalized epidemic) had higher odds of AHD compared with females, we observed a significant decrease in AHD in both sexes over time. Haiti has broad public health interventions to promote HIV prevention and care [28], including pre‐exposure prophylaxis interventions [29], and receives external support, which allows for wider diagnostic campaigns and ART treatment programmes [27, 30]. In our study, we found a median time of 0 days between HIV diagnoses and enrolment in care in Haiti, supporting their early access to care and a lower proportion of AHD in their cohort. Furthermore, the number of new HIV acquisitions has decreased between 2010 and 2022 [27], which may be related to their progress in primary prevention [31, 32] and, in consequence, explain the decreasing prevalence of AHD at enrolment we observed.

In concentrated epidemic sites, we observed that HMs and females had higher odds of AHD at enrolment compared with males with male‐to‐male sexual contact. The males with male‐to‐male sexual contact group was younger and more educated compared with HMs and females, possibly resulting in more health literacy, greater awareness of HIV acquisition, earlier access to prevention and care interventions, and better clinical outcomes [33]. We observed the same trends after including the unknown HIV acquisition group in the model. Consistent with other studies, older age and low‐level education at enrolment were associated with higher odds of AHD in all the sex‐at‐birth/HIV acquisition groups in concentrated epidemic settings [17, 22, 34].

Groups with low perceived risk in concentrated epidemic settings, particularly females (especially after childbearing age) and HMs, may face important barriers to HIV testing and screening [7, 35]. Because these populations are not traditionally considered key populations, they may have lower awareness and reduced access to HIV testing services. Therefore, the sex disparities in AHD we observed may reflect persistent barriers to HIV testing, limited tools to optimize testing services [36], and the lack of acceptable, feasible and effective preventive interventions, such as pre‐exposure prophylaxis [37].

The proportion of individuals presenting with AHD in concentrated epidemics was higher than in the generalized epidemic setting. Among females, this difference was nearly 10 percentage points. Haiti has one of the oldest HIV/AIDS epidemics outside of sub‐Saharan Africa [38], in addition to structural challenges, including poverty, institutional weakness, inequality, racism, limited public health responses, and stigma embedded in laws and policies, contributing to higher HIV prevalence [39, 40]. Moreover, Haiti receives international funding [41] as opposed to many Latin American countries, which tend to be domestically funded, with limited international support, which may constrain financing and lead to prioritization of key populations, often overlooking vulnerable groups such as females [42].

Another possible explanation for the differences of AHD prevalence by epidemic type and sex disparities is HIV‐related stigma that negatively affects every step of the HIV continuum [43], and health‐related outcomes [44]. Specifically, stigma is associated with lower levels of HIV testing [45] and increased late presentation [46]. Countries with lower HIV prevalence tend to exhibit higher levels of HIV‐related stigma, partly explained by limited HIV‐related knowledge, reduced contact with PWH, lack of normalization of HIV and weaker structural responses [45, 47, 48]. Moreover, depression, rural residence, female sex and low socioeconomic status are significantly associated with higher levels of HIV‐related stigma [49]. Consequently, stigma and other structural factors affecting females may increase their risk of delaying diagnosis, particularly in settings with concentrated epidemics.

Our study has several limitations. The cohort includes seven mostly urban referral sites and may not represent other countries, rural populations or sites outside CCASAnet. Some sites in concentrated epidemics are not part of the public system, and administrative barriers related to social or private insurance may discourage HIV testing and delay the enrolment into care. Differences in CD4 measurement frequency, availability at HIV diagnosis and reporting of AIDS‐defining events may have led to underestimation of AHD, particularly in Haiti. Additionally, declining availability of CD4 data over time may bias CD4‐based findings [20, 21]. To improve AHD estimation, we expanded the CD4 measurement window and included AIDS diagnosis regardless of CD4 availability. Despite the limitations, this is one of the largest longitudinal cohorts in the region with ongoing data quality audits [50]. It is also one of the few studies with stratified analyses by epidemic type, sex at birth and HIV acquisition, contributing to the understanding of specific sex‐ and gender‐based health disparities [51].

## 5 Conclusions

Females and HMs in concentrated epidemics in Latin America are at a higher risk of initiating HIV care with AHD compared with males with male‐to‐male sexual contact. Their sociodemographic characteristics at enrolment, including older age and lower educational attainment, likely reflect limited awareness of HIV risk among both individuals and healthcare providers. These findings highlight the need for targeted context‐specific interventions that go beyond health education to improve risk perception, promote earlier HIV diagnosis and facilitate timely linkage to care. Importantly, such interventions should also address broader structural barriers, including gender norms and inequities. Addressing these gaps will be critical to improve HIV outcomes in the region.

## Author Contributions

YC‐V, JM‐C, LG‐T and BCR were responsible for the study concept and study design. BCR supervised the implementation of the study. YC‐V provided methodological expertise. YC‐V, JM‐C and BCR conducted the search and review of the literature. BCR, CC, CPC, DMM, PML, JA‐P, MTL, EG and VR were responsible for the acquisition of the data. YC‐V was responsible for the interpretation of the data. YC‐V, JM‐C, LG‐T, CC, CPC, DMM, PML, JA‐P, MTL, EG, VR, JLC, PFR and BCR made critical revision for important intellectual content. YC‐V, JM‐C, LG‐T and BCR drafted the manuscript. YC‐V, JM‐C, LG‐T and BCR read and commented all previous versions of the manuscript. YC‐V, JM‐C, LG‐T, CC, CPC, DMM, PML, JA‐P, MTL, EG, VR, JLC, PFR and BCR read and gave their final approval of the final version of the manuscript.

## Funding

This work was supported by the NIH‐funded Caribbean, Central and South America network for HIV epidemiology (CCASAnet), a member cohort of the International Epidemiologic Databases to Evaluate AIDS (leDEA) (U01AI069923). This award is funded by the following institutes: the National Institute of Allergy and Infectious Diseases (NIAID), the Eunice Kennedy Shriver National Institute of Child Health and Human Development (NICHD), the National Cancer Institute (NCI), the National Institute of Mental Health (NIMH), the National Institute on Drug Abuse (NIDA), the National Heart, Lung, and Blood Institute (NHLBI), the National Institute on Alcohol Abuse and Alcoholism (NIAAA), the National Institute of Diabetes and Digestive and Kidney Diseases (NIDDK), the Fogarty International Center (FIC) and the National Library of Medicine (NLM).

## Conflicts of Interest

The authors declare that they have no conflicts of interest.

## Disclaimer

The content is solely the responsibility of the authors and does not necessarily represent the official views of the National Institutes of Health.

External requests should be directed to the CCASAnet Program Coordinator, Ms. Hilary Vansell (hilary.vansell@vumc.org).

## Supporting information



Supporting Information File 1: Supplementary Material.
**Table S1**. Sensitivity analysis for concentrated epidemic settings: Associated characteristics with advanced HIV disease at enrolment in care in the study period by group of sex‐at‐birth/HIV acquisition in concentrated epidemics.
**Table S2**. Sensitivity analysis for concentrated epidemic settings: Associated characteristics with advanced HIV disease at enrolment in care in the study period by group of sex‐at‐birth/HIV acquisition in concentrated epidemics (Stratified models, unknown mode of HIV acquisition as a category).
**Table S3**. Sensitivity analysis for concentrated epidemic settings: Associated characteristics with advanced HIV disease at enrolment in care in the study period by group of sex‐at‐birth/HIV acquisition in concentrated epidemics (Stratified models, imputed dataset).
**Table S4**. Description of the models fitted in tables and figures of the manuscript and Supplementary Material. The number of participants included in each model is reported in the cells.

**Figure S1**. Sensitivity analysis for concentrated epidemic settings: Adjusted probability of advanced HIV disease (AHD) at enrolment over time by sex at birth and HIV acquisition groups and epidemic setting. Top plot: Model without imputation of sex at birth and HIV acquisition groups. Bottom plot: Model with imputation of sex at birth and HIV acquisition groups.

**Figure S1**. Sensitivity analysis for concentrated epidemic settings (Stratified by group): Adjusted probability of advanced HIV disease (AHD) at enrolment over time by sex at birth and HIV acquisition groups and epidemic setting. Top plot: Model without imputation of sex at birth and HIV acquisition groups including Unknown as a category. Bottom plot: Model with imputation of sex at birth and HIV acquisition groups.

## Data Availability

Data are made available to all authors and external collaborators upon reasonable request, in accordance with internal scientific review guidelines (https://www.iedea.org/regions/ccasanet/ https://www.iedea.org/wp‐content/uploads/2024/04/Final‐IeDEA‐global‐SOP‐interim‐revision_15‐March‐2024.pdf).
