# Supplementary material for: Sex Differences in Advanced HIV Disease at Presentation in Care Among People Living With HIV in Latin America and the Caribbean
Source: J Int AIDS Soc. 2026 Aug 9;29(8):e70161. doi: 10.1002/jia2.70161 (PMC13454556; doi:10.1002/jia2.70161)
Supplement: Supplementary file 1 [file JIA2-29-e70161-s003.docx]

***Supplementary Material***

**Figure S1. Sensitivity analysis for concentrated epidemic settings (Stratified by group):** Adjusted probability of advanced HIV disease (AHD) at enrollment over time by sex at birth and HIV acquisition groups and epidemic setting. Top plot: model without imputation of sex at birth and HIV acquisition groups including Unknow as a category. Bottom plot: model with imputation of sex at birth and HIV acquisition groups.

***Note:*** Stratified models by group of Sex at birth and HIV acquisition, models 10-16 in Supplementary Table S4.

**Table S1**. **Sensitivity analysis for concentrated epidemic settings:** associated characteristics with advanced HIV disease at enrollment in care in the study period by group of sex-at-birth/HIV acquisition in concentrated epidemics.

| **Variable** | | **Group** | **Model without imputation of sex-at-birth/HIV acquisition group** | **Model with imputation of sex-at-birth/HIV acquisition group** |
| --- | --- | --- | --- | --- |
|  | Males with male-to-male sexual contact | | 1 | 1 |
| **Sex at birth** | Females | | 1.12 (1.04 – 1.20) | 1.15 (1.07 – 1.25) |
|  | Heterosexual males | | 2.00 (1.87 – 2.15) | 1.96 ( 1.81 – 2.11) |
|  | Unknown | | 1.23 (1.11–-1.35) | NA |
| **Age at enrollment** | 30 | | 1 | 1 |
|  | 40 | | 1.44 (1.40 – 1.49) | 1.44 (1.40 – 1.49) |
|  | 50 | | 1.44 (1.37 – 1.52) | 1.44 (1.37 – 1.52) |
| **Education level** | Less than primary | | 1.34 (1.23 – 1.46) | 1.33 (1.23 – 1.45) |
|  | Secondary | | 1 | 1 |
|  | University | | 0.74 (0.69 – 0.78) | 0.74 (0.69 – 0.79) |
|  | Unknown | | 0.84 (0.77-0.92) | 0.84 (0.77 – 0.92) |
| **Calendar year of enrollment** | 2003 | | 1 | 1 |
|  | 2010 | | 0.72 (0.67 – 0.78) | 0.73 (0.67 – 0.79) |
|  | 2022 | | 0.36 (0.32 – 0.39) | 0.36 (0.33 – 0.39) |

***Note****:* Overall associations results from a logistic model without sex-at-birth/HIV acquisition stratification. Models 8,9 in the Supplementary Table S4.

**Table S2**. **Sensitivity analysis for concentrated epidemic settings:** associated characteristics with advanced HIV disease at enrollment in care in the study period by group of sex-at-birth/HIV acquisition in concentrated epidemics (Stratified models, unknown mode of HIV acquisition as a category).

| **Variable** | **Group** | **Stratified by sex-at-birth/HIV acquisition group models** | | | |
| --- | --- | --- | --- | --- | --- |
|  |  | **Females** | **Heterosexual males** | **Males with male-to-male sexual contact** | **Unknown** |
| **Age at enrollment** | 30 | 1 | 1 | 1 | 1 |
|  | 40 | 1.47 (1.37 – 1.57) | 1.29 (1.19 – 1.45) | 1.44 (1.38 -1.49) | 1.30 (1.14 – 1.48) |
|  | 50 | 1.56 (1.39 – 1.75) | 1.31 (1.19 – 1.39) | 1.45 (1.31 – 1.59) | 1.28 (1.10 – 1.50) |
| **Education level** | Less than primary | 1.37 (1.18 – 1.59) | 1.28 (1.09 – 1.50) | 1.71 (1.46 – 2.01) | 3.03 (2.37 – 3.88) |
|  | Secondary | 1 | 1 | 1.33 (1.23 – 1.44) | 2.15 (1.75 – 2.63) |
|  | University | 0.81 (0.68 – 0.96) | 0.71 (0.62 – 0.82) | 1 | 0.88 (0.62 – 1.19) |
|  | Unknown | 1.22 (1.00 – 1.49) | 0.94 (0.76 – 1.17) | 1.13 (0.99 – 1.29) | 1 |
| **Calendar year of enrollment** | 2003 | 1 | 1 | 1 | 1 |
|  | 2010 | 0.66 (0.55 – 0.79) | 0.99 (0.82 – 1.20) | 0.70 (0.63 – 0.78) | 0.55 (0.41 – 0.67) |
|  | 2022 | 0.57 (0.46 – 0.79) | 0.47 (0.38 – 0.58) | 0.22 (0.19 – 0.25) | 0.73 (0.56 – 0.96) |

***Note****:* Overall associations results from logistic models by sex-at-birth/HIV acquisition strata. Models 10-13 in the Supplementary Table S4.

**Table S3**. **Sensitivity analysis for concentrated epidemic settings:** associated characteristics with advanced HIV disease at enrollment in care in the study period by group of sex-at-birth/HIV acquisition in concentrated epidemics (Stratified models, imputed dataset).

| **Variable** | **Group** | **Stratified by sex-at-birth/HIV acquisition group models^a^** | | |
| --- | --- | --- | --- | --- |
|  |  | **Females** | **Heterosexual males** | **Males with male-to-male sexual contact** |
| **Age at enrollment** | 30 | 1 | 1 | 1 |
|  | 40 | 1.43 (1.34 – 1.53) | 1.29 (1.19 – 1.39) | 1.43 (1.38 -1.49) |
|  | 50 | 1.53 (1.37 – 1.69) | 1.29 (1.17 – 1.42) | 1.40 (1.28 – 1.53) |
| **Education level** | Less than primary | 1.34 (1.16 – 1.54) | 1.33 (1.13 – 1.55) | 1.34 (1.15 – 1.56) |
|  | Secondary | 1 | 1 | 1 |
|  | University | 0.79 (0.67 – 0.94) | 0.71 (0.62 – 0.82) | 0.73 (0.67 – 0.79) |
|  | Unknown | 1.04 (0.87 – 1.24) | 0.71 (0.59 – 0.85) | 0.79 (0.69 – 0.89) |
| **Calendar year of enrollment** | 2003 | 1 | 1 | 1 |
|  | 2010 | 0.61 (0.51 – 0.72) | 0.96 (0.81 – 1.15) | 0.68 (0.62 – 0.75) |
|  | 2022 | 0.61 (0.50 – 0.74) | 0.48 (0.39 – 0.58) | 0.24 (0.21 – 0.28) |

***Note****:* Overall associations results from logistic models by sex-at-birth/HIV acquisition strata. Models 14-16 in the Supplementary Table S4.

**Table S4.** **Description of the models fitted in tables and figures of the manuscript and supplementary material.** The number of participants included in each model is reported in the cells.

| **Model** | | **M1** | **M2** | **M3** | **M4** | **M5** | **M6** | **M7** | **M8** | **M9** | **M10** | **M11** | **M12** | **M13** | **M14** | **M15** | **M16** |
| --- | --- | --- | --- | --- | --- | --- | --- | --- | --- | --- | --- | --- | --- | --- | --- | --- | --- |
| **Location**  Table, Figure | | T2 | T2,  Fig3 | T2,  Fig3 | T3, | T3,  Fig3 | T3,  Fig3 | T3,  Fig3 | TS1 | TS1 | TS2,  FigS1 top | TS2,  FigS1 top | TS2,  FigS1 top | TS2,  FigS1 top | TS3,  FigS1 bottom | TS3,  FigS1 bottom | TS3,  FigS1 bottom |
| **Epidemic Setting** | | **Generalized** | | | **Concentrated** | | | | | | | | | | | | |
|  | **Type of model** | Marginal | Stratified | | Marginal | Stratified | | | Marginal | | Stratified  (Without imputation) | | | | Stratified (With imputation) | | |
| **Primary exposure (strata)** |  |  |  |  |  |  |  |  |  |  |  |  |  |  |  |  |  |
| Sex | | 24424 |  |  |  |  |  |  |  |  |  |  |  |  |  |  |  |
| Calendar year-by-sex at birth (Females only) | |  | 14141 |  |  |  |  |  |  |  |  |  |  |  |  |  |  |
| Calendar year-by-sex at birth (Males only) | |  |  | 10283 |  |  |  |  |  |  |  |  |  |  |  |  |  |
| Sex-by-HIV Acquisition Group | |  |  |  | 23124 |  |  |  |  |  |  |  |  |  |  |  |  |
| Calendar year Sex-by-HIV Acquisition Group (Females Only) | |  |  |  |  | 4637 |  |  |  |  |  |  |  |  |  |  |  |
| Calendar year Sex-by-HIV Acquisition Group (Hetero Males Only) | |  |  |  |  |  | 5055 |  |  |  |  |  |  |  |  |  |  |
| Calendar year Sex-by-HIV Acquisition Group (Males with male-to-male sexual contact Only) | |  |  |  |  |  |  | 13432 |  |  |  |  |  |  |  |  |  |
| Sex-by-HIV Acquisition Group (Includes unknown category) | |  |  |  |  |  |  |  | 25456 |  |  |  |  |  |  |  |  |
| Sex-by-HIV Acquisition Group (Imputed) | |  |  |  |  |  |  |  |  | 25456 |  |  |  |  |  |  |  |
| Calendar year Sex-by-HIV Acquisition Group (Females Only) | |  |  |  |  |  |  |  |  |  | 4637 |  |  |  |  |  |  |
| Calendar year Sex-by-HIV Acquisition Group (Hetero Males Only) | |  |  |  |  |  |  |  |  |  |  | 5055 |  |  |  |  |  |
| Calendar year Sex-by-HIV Acquisition Group ((Males with male-to-male sexual contact Only) | |  |  |  |  |  |  |  |  |  |  |  | 13432 |  |  |  |  |
| Calendar year Sex-by-HIV Acquisition Group (Unknown Only) | |  |  |  |  |  |  |  |  |  |  |  |  | 2332 |  |  |  |
| Calendar year Sex-by-HIV Acquisition Group (Females Only*) | |  |  |  |  |  |  |  |  |  |  |  |  |  | 5178 |  |  |
| Calendar year Sex-by-HIV Acquisition Group (Hetero Males Only*) | |  |  |  |  |  |  |  |  |  |  |  |  |  |  | 5691 |  |
| Calendar year Sex-by-HIV Acquisition Group ((Males with male-to-male sexual contact Only *) | |  |  |  |  |  |  |  |  |  |  |  |  |  |  |  | 14587 |
| ***Note:*** *All models adjusted for Age, Education, & Calendar Year. * The strata include the number of one of the replication datasets from imputation process* | | | | | | | | | | | | | | | | | |
